# Coxsackievirus B3 elicits a sex-specific CD8^+^ T cell response which protects female mice

**DOI:** 10.1371/journal.ppat.1011465

**Published:** 2023-09-05

**Authors:** Adeeba H. Dhalech, Stephanie A. Condotta, Aryamav Pattnaik, Caleb Corn, Martin J. Richer, Christopher M. Robinson

**Affiliations:** Department of Microbiology and Immunology, Indiana University School of Medicine, Indianapolis, Indiana, United States of America; Johns Hopkins Bloomberg School of Public Health, UNITED STATES

## Abstract

Sex is a significant contributor to the outcome of human infections. Males are frequently more susceptible to viral, bacterial, and fungal infections, often attributed to weaker immune responses. In contrast, a heightened immune response in females enables better pathogen elimination but leaves females more predisposed to autoimmune diseases. Unfortunately, the underlying basis for sex-specific immune responses remains poorly understood. Here, we show a sex difference in the CD8^+^ T cell response to an enteric virus, Coxsackievirus B3 (CVB3). We found that CVB3 induced expansion of CD8^+^ T cells in female mice but not in male mice. CVB3 also increased the proportion and number of CD11a^hi^CD62L^lo^ CD8^+^ T cells in female mice, indicative of activation. This response was independent of the inoculation route and type I interferon. Using a recombinant CVB3 virus expressing a model CD8^+^ T cell epitope, we found that the expansion of CD8^+^ T cells in females is viral-specific and not due to bystander activation. Finally, the depletion of CD8^+^ T cells, prior to infection, led to enhanced mortality, indicating that CD8^+^ T cells are protective against CVB3 in female mice. These data demonstrate that CVB3 induces a CD8^+^ T cell response in female mice and highlight the importance of sex-specific immune responses to viral pathogens.

## Author summary

Despite the impact of enteric viruses on human health, many enteric viruses lack an approved vaccine. Strategies for vaccine development are primarily focused on the induction of neutralizing antibodies; however, previous studies indicate that antibodies may not contribute to protection against some enteric viruses, including Coxsackievirus B3 (CVB3). Therefore, the mechanism(s) contributing to a protective immune response against CVB3 is unclear. Prior data have indicated that CVB3 infection of mice fails to induce a CD8^+^ T cell response. However, these conclusions were based primarily on the infection of male mice. Here we make the novel observation that CVB3 induces the expansion of antigen-experienced CD8^+^ T cells in female mice but not in male mice. The CD8^+^ T cells in female mice have an activated phenotype following infection, and we demonstrate that these T cells protect female mice from CVB3-induced mortality. Overall, our findings emphasize the importance of evaluating sex differences in the immune response to viral pathogens and may have implications for future T cell vaccine strategies.

## Introduction

Sex is a significant contributor to the outcome of various viral and bacterial infections. These sex-dependent outcomes are likely due to differences in the immune response between men and women. Previous studies have demonstrated that a heightened immune response in women promotes enhanced pathogen clearance compared to men [1]. This heightened immune response is not without consequences, as females often experience more severe symptoms following infection and are more predisposed to autoimmune diseases. Moreover, sex-specific immunity also influences vaccine responses [2]. Therefore, there is a critical need to understand the mechanism(s) that influence sex-dependent immune responses to pathogens.

T cells play an essential role in the host immune response to viral pathogens, and memory T cells contribute to vaccine-induced immunity [3–5]. Several studies have demonstrated differences in the number and proportion of T cells in men and women. Women tend to have higher CD4^+^ T cell counts and a higher CD4/CD8 ratio than men [6]. Activation of T cells is also enhanced in women. Upon PMA-ionomycin stimulation, CD4^+^ T cells from females exhibit increased upregulation of TNFα, IFN-γ, and IL-17 [7]. Similarly, activated female CD8^+^ T cells show enhanced upregulation of antiviral and proinflammatory genes compared to their male counterparts [8]. Overall, these data indicate that sex differences in T cell responses may play a crucial role in the outcome of intracellular infections. However, questions remain how sex differences in the T cell response impact specific viral infections.

Coxsackievirus B3 (CVB3) is a small, non-enveloped RNA virus in the *Picornaviridae* family. In humans, CVB3 is a primary cause of viral myocarditis. A sex bias in viral myocarditis has been observed where males are twice as likely to have severe sequela compared to females. This sex bias is also observed in the murine models of CVB3 [9, 10]. In mice, the immune response to CVB3 has been implicated as a critical contributor to viral myocarditis. Acute CVB3-induced myocarditis is characterized by inflammatory infiltration of immune cells, including CD4^+^ and CD8^+^ T cells [11,12]. Several studies have shown that these T cells contribute to CVB3 clearance but can also facilitate disease following infection [13]. Differences in the Th1 and Th2 CD4^+^ T cell responses between males and females influence myocarditis in mice [14,15]. Further, γδ T cells and sex hormones can tip the balance of this CD4^+^ T cell response to either promote or limit disease [16–19]. Similarly, CD8^+^ T cells have also been shown to play dual roles in myocarditis. Depleting CD8^+^ T cells leads to increased viral titers in mice; however, CVB3 infection of CD8^+^ T cell-deficient, beta-2 microglobulin knockout mice are more resistant to the development of myocarditis [13]. Unfortunately, efforts to elucidate CVB3-specific CD8^+^ T cell response have been hampered due to the limited ability to induce CD8^+^ T cell expansion *in vivo* [20–23]. Thus, the role of CD8^+^ T cells during CVB3 infections is still unclear.

CVB3 is a member of the enterovirus group that is transmitted through the fecal-oral route and initiates infection in the gastrointestinal tract. Previously we established an oral inoculation mouse model for CVB3 to recapitulate a natural route of infection through the intestine [24]. This model uses C57BL/6 *Ifnar*^*-/-*^ (deficient for interferon α/β receptor) mice to facilitate viral replication in the intestine [24,25]. We recently determined that testosterone, the primary sex hormone in males, can promote intestinal CVB3 replication and viral dissemination in male and female mice [26]. Further, gonadectomy of male mice to deplete endogenous testosterone completely protected males from CVB3-induced lethality. In contrast, testosterone and ovariectomy of female mice do not alter the susceptibility to CVB3-induced mortality [24,26]. Therefore, these data suggest sex hormones contribute to intestinal viral replication and dissemination; however, other unknown immune system components protect female mice from CVB3-associated lethality.

In this study, we sought to evaluate the CVB3-specific immune response in orally inoculated male and female mice. We found that CVB3 elicited a significant expansion of CD4^+^ and CD8^+^ T cells in female mice but not in male mice. We further evaluated the CD4^+^ and CD8^+^ T cell response in female mice for activation and found that CD8^+^ T cells from female mice presented with an activated phenotype. We also found that the expansion CD8^+^ T cells from female mice are independent of the route of inoculation and type I IFN. Using a recombinant CVB3 strain (rCVB3-GP_33_) that has LCMV- specific CD8^+^ cytotoxic T lymphocyte GP_33-41_ epitope integrated into its polyprotein [20,21,27] we found that CD8^+^ T cell expansion was driven by viral antigen rather than bystander activation. Finally, we found that CD8^+^ T cells are critical for protection from CVB3 infection in female mice as depletion of CD8^+^ T cells significantly increases mortality. These findings indicate a novel, protective CD8^+^ T cell response to CVB3 in female mice and further emphasize the inclusion of sex as a variable in the immune response to viral pathogens.

## Results

### Expansion of CD4^+^ and CD8^+^ T cells occurs in female mice but not in male mice following oral inoculation with CVB3

**Fig 1:**
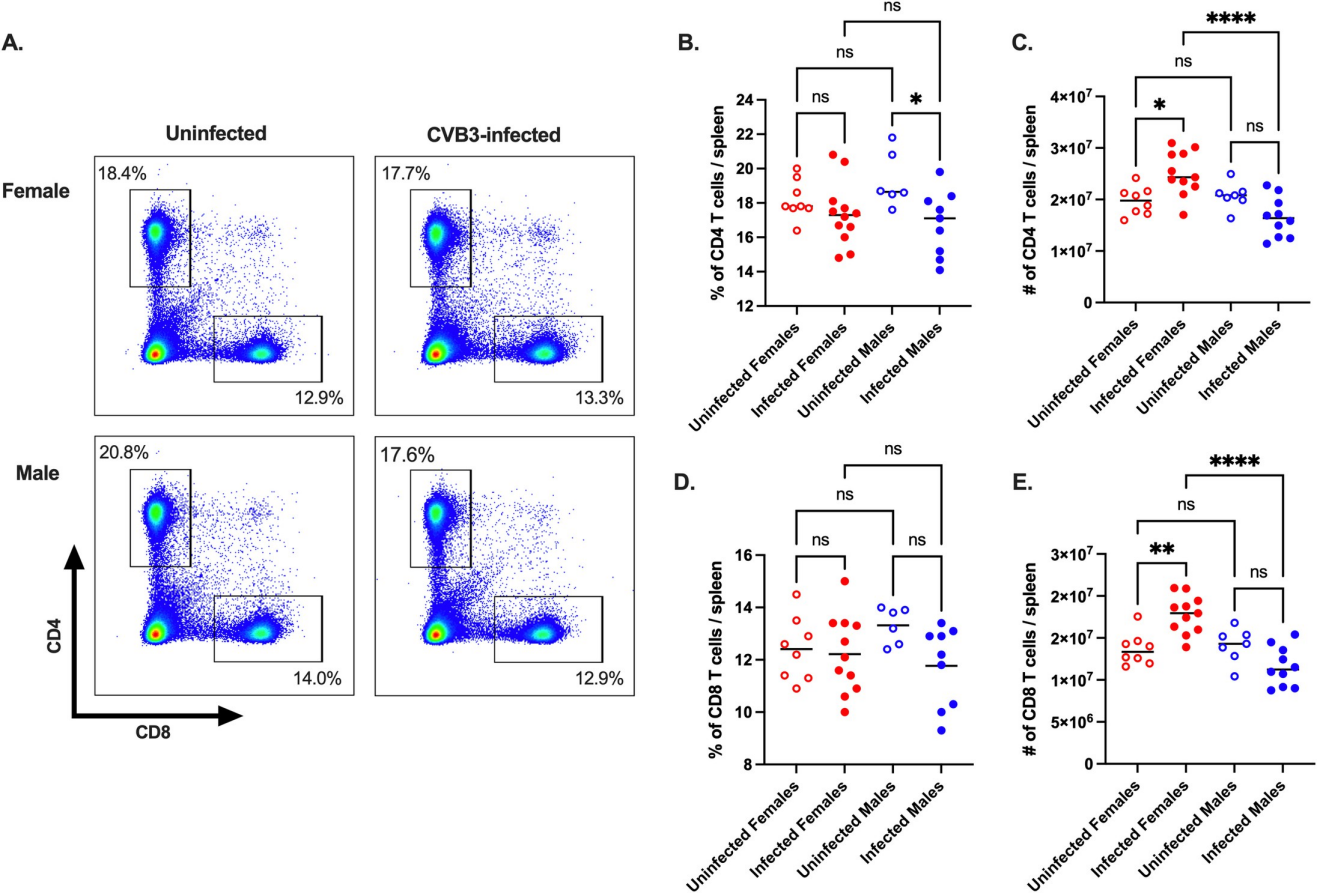
The CD4^+^ and CD8^+^ T cell response in CVB3-infected male and female *Ifnar*^*-/-*^ mice. Male and female *Ifnar*^*-/-*^ mice were orally inoculated with 5x10^7^ PFUs of CVB3. The spleen was harvested at 5dpi, and splenocytes were processed for analysis by flow cytometry. (A) Representative flow cytometry plots of T cells gated on CD4^+^ and CD8^+^ T cell expression in uninfected and infected male and female mice. The frequency (B) and number (C) of splenic CD4^+^ T cells 5dpi. The frequency (D) and number (C) of splenic CD8^+^ T cells 5dpi. Data points in the scatter plots represent individual mice, with lines representing the mean from two experiments. *p<0.05, **p<0.01, ****p<0.0001, One-way ANOVA.

We previously found that female *Ifnar*^*-/-*^ mice are protected against CVB3-induced lethality, and this protection did not correlate to intestinal replication and dissemination [26]. Therefore, we hypothesized that sex differences in the immune response might offer females protection against CVB3 following oral inoculation. To determine immune correlates of protection, we orally inoculated male and female C57BL/6 *Ifnar*^*-/-*^ mice with 5x10^7^ PFUs of CVB3-Nancy, collected the spleens at 5 days post-inoculation (dpi), and analyzed splenocytes by flow cytometry. We chose to examine immune cells at 5dpi because we have previously shown that mortality in male mice typically begins at this time point [24,26]. At 5dpi, while we observed higher numbers of splenic neutrophils and macrophages in uninfected male mice compared to female mice, we found no difference in the frequency and numbers of B cells, neutrophils, macrophages, and dendritic cells between uninfected and infected mice of either sex (S1 Fig). Contrary to other immune cells, we observed a significant decrease in the frequency of CD4^+^ T cells in infected male mice compared to uninfected male mice (Fig 1A and 1B). The overall number of splenic CD4^+^ T cells in CVB3-infected male mice was also reduced compared to uninfected male mice; however, this did not reach statistical significance (Fig 1C). Similarly, the frequency and number of CD8^+^ T cells in infected male mice were reduced compared to uninfected male mice, but this was not statistically significant (Fig 1D and 1E). In female mice, we found no significant increase in the frequency of CD4^+^ and CD8^+^ T cells in infected or uninfected groups. However, in contrast to males, we found a significant increase in the number of splenic CD4^+^ and CD8^+^ T cells in CVB3-infected female mice compared to uninfected female mice (Fig 1C and 1E). Moreover, the number of CD4^+^ and CD8^+^ T cells was significantly higher in CVB3-infected female mice compared to CVB3-infected male mice (Fig 1C and 1E). Additionally, we found that the increased T cell expansion in female mice was not due to higher viral loads in the spleen and peripheral tissues. Following oral inoculation, we observed that male mice had significantly higher viral tissue titers in the heart, kidney, spleen, and pancreas compared to female mice (S2 Fig). Overall, these data indicate that female mice have increased numbers of splenic T cells following CVB3 infection.

### Activation of CD8^+^ T cells occurs in the spleen and mesenteric lymph nodes of CVB3-infected female mice

**Fig 2:**
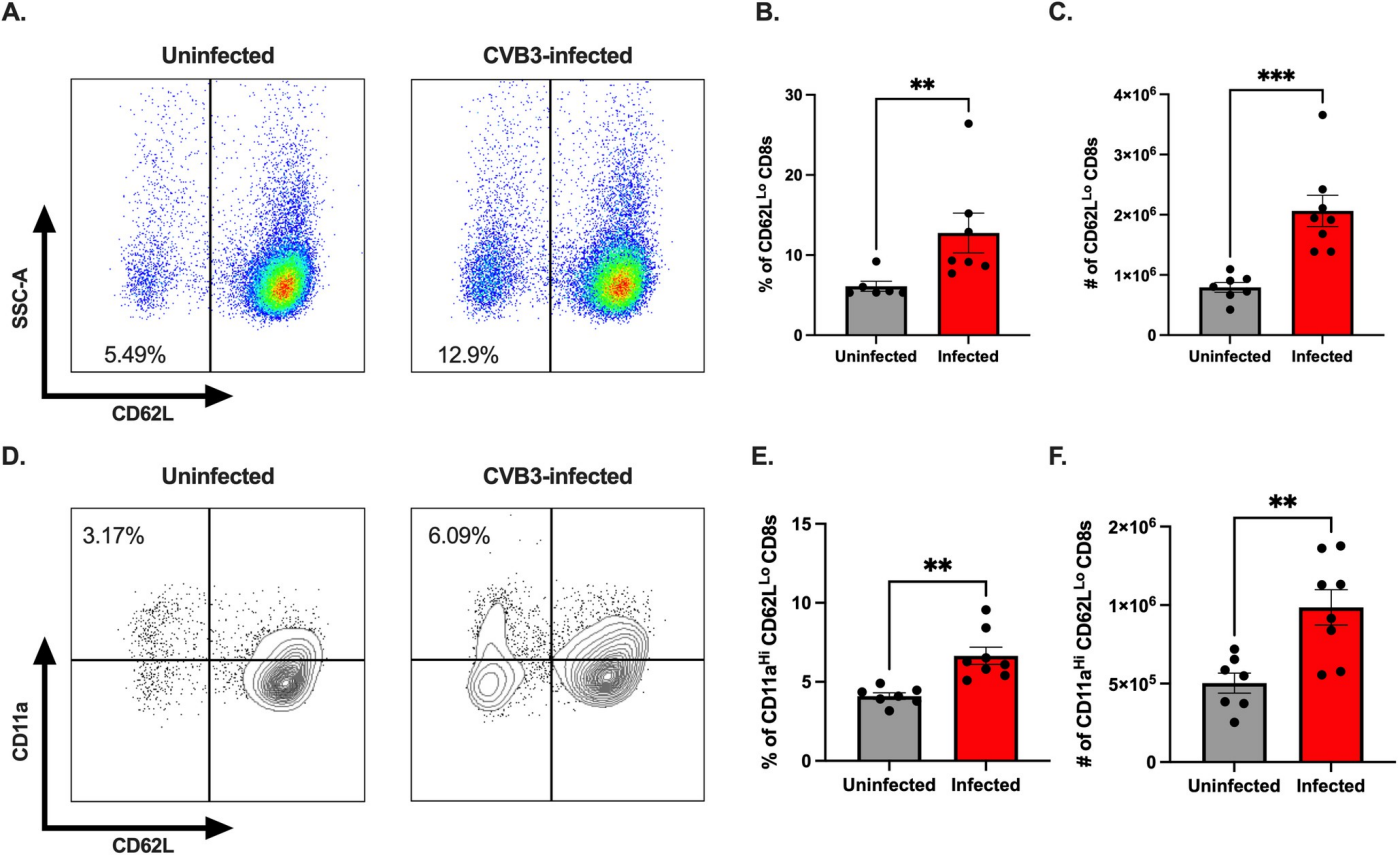
CVB3 induces an expansion of activated CD8^+^ T cells in female *Ifnar*^*-/-*^ mice. (A) Representative flow cytometry plots of CD62L expression on CD8^+^ T cells in uninfected and infected female *Ifnar*^*-/-*^ mice. The frequency (B) and number (C) of CD62L^lo^ CD8^+^ T cells in the spleen 5dpi in *Ifnar*^*-/-*^ mice. (D) Representative flow cytometry plots of CD11a and CD62L expression gated on CD8^+^ T cells in uninfected and infected female *Ifnar*^*-/-*^ mice. The frequency (E) and number (F) of CD11a^hi^CD62L^lo^ CD8^+^ T cells in female mice 5dpi. All data are from two independent experiments with n = 7–8 mice per group and are shown as mean ± SEM. *p<0.05, **p<0.01, ***p<0.001, unpaired t-test.

Previously, we found that CD8^+^ T cell activation was limited in male mice due to testosterone [26]. Based on the increase in the numbers of CD4^+^ and CD8^+^ T cells in infected female mice, we hypothesized that T cells from female mice are activated following CVB3 infection. First, we assessed CD8^+^ T cell activation by the expression of CD62L. Naïve CD8^+^ T cells are CD62L^hi^, and activated CD8^+^ T cells differentiate into effector subtypes during acute infections. During this effector phase, CD62L is downregulated [28–30]. Following CVB3 inoculation, we observed a significant increase in the frequency and number of CD62L^lo^ CD8^+^ T cells in infected female mice compared to uninfected female mice (Fig 2A–2C). Next, we investigated the expression of the integrin molecule CD11a on CD62L^lo^ CD8^+^ T cells. Upregulation of CD11a can also distinguish naïve CD8^+^ T cells from effector and memory CD8^+^ T cells [28]. We found that infection with CVB3 significantly increased the frequency and number of CD11a^hi^CD62L^lo^ CD8^+^ T cells in female mice (Fig 2D–2F). Taken together, these data indicate that CVB3 alters the frequency and number of CD62L^lo^ and CD11a^hi^CD62L^lo^ CD8^+^ T cells in female mice, indicative of CD8^+^ T cell activation.

**Fig 3:**
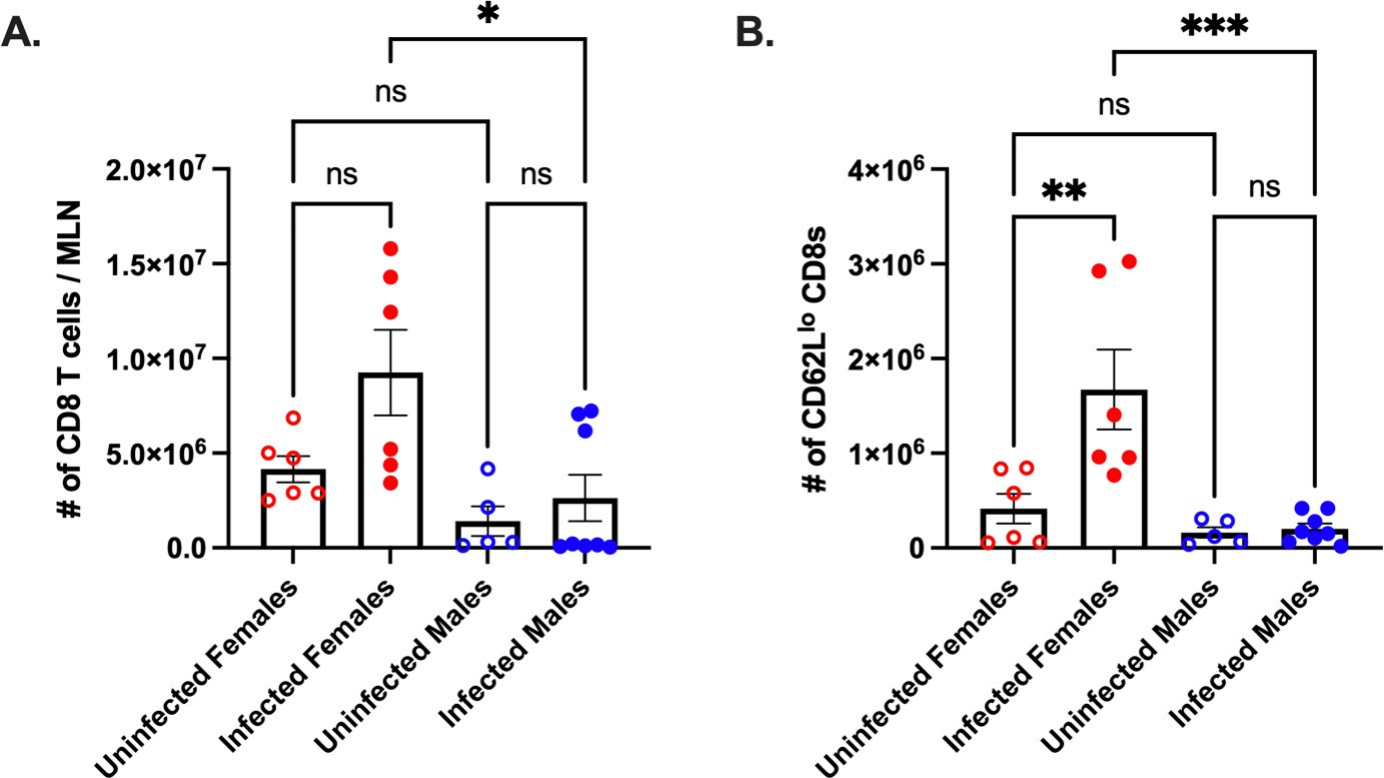
CVB3 induces expansion of activated CD8^+^ T cells in the mesenteric lymph nodes of female *Ifnar*^*-/-*^ mice. (A) The number of mesenteric lymph node CD8^+^ T cells 5dpi. (B) The number of CD62L^lo^ CD8^+^ T cells in the mesenteric lymph node 5dpi in *Ifnar*^*-/-*^ mice. All data are from two independent experiments with n = 5–8 mice per group and are shown as mean ± SEM. *p<0.05, **p<0.01, ***p<0.001, One-way ANOVA.

Next, since CVB3 initiates infection in the intestine, we examined if CD8^+^ T cells are activated in the local lymph node following oral inoculation. Male and female *Ifnar*^*-/-*^ mice were orally inoculated with CVB3, and the mesenteric lymph nodes (MLNs) were harvested at 5dpi. We observed similar numbers of CD8^+^ T cells in both uninfected and infected male mice (Fig 3A). However, while there was a trending increase in the number of CD8^+^ T cells in infected female mice compared to uninfected female mice (p = 0.09), we found a significant increase in the number of MLN CD8^+^ T cells from infected female mice compared to infected male mice (Fig 3A). Further, we observed a significant increase in CD62L^lo^ CD8^+^ T cells from infected female mice compared to both uninfected female mice and infected male mice (Fig 3B). Overall, these data indicate that like the spleen, CVB3 infection increases the number of CD62L^lo^ CD8^+^ T cells in the MLNs of infected female mice, but not in male mice, providing evidence of enhanced CD8^+^ T cell activation in female mice.

### Expansion of antigen-experienced CD8^+^ T cells occurs in CVB3-infected female mice but not in male mice

**Fig 4:**
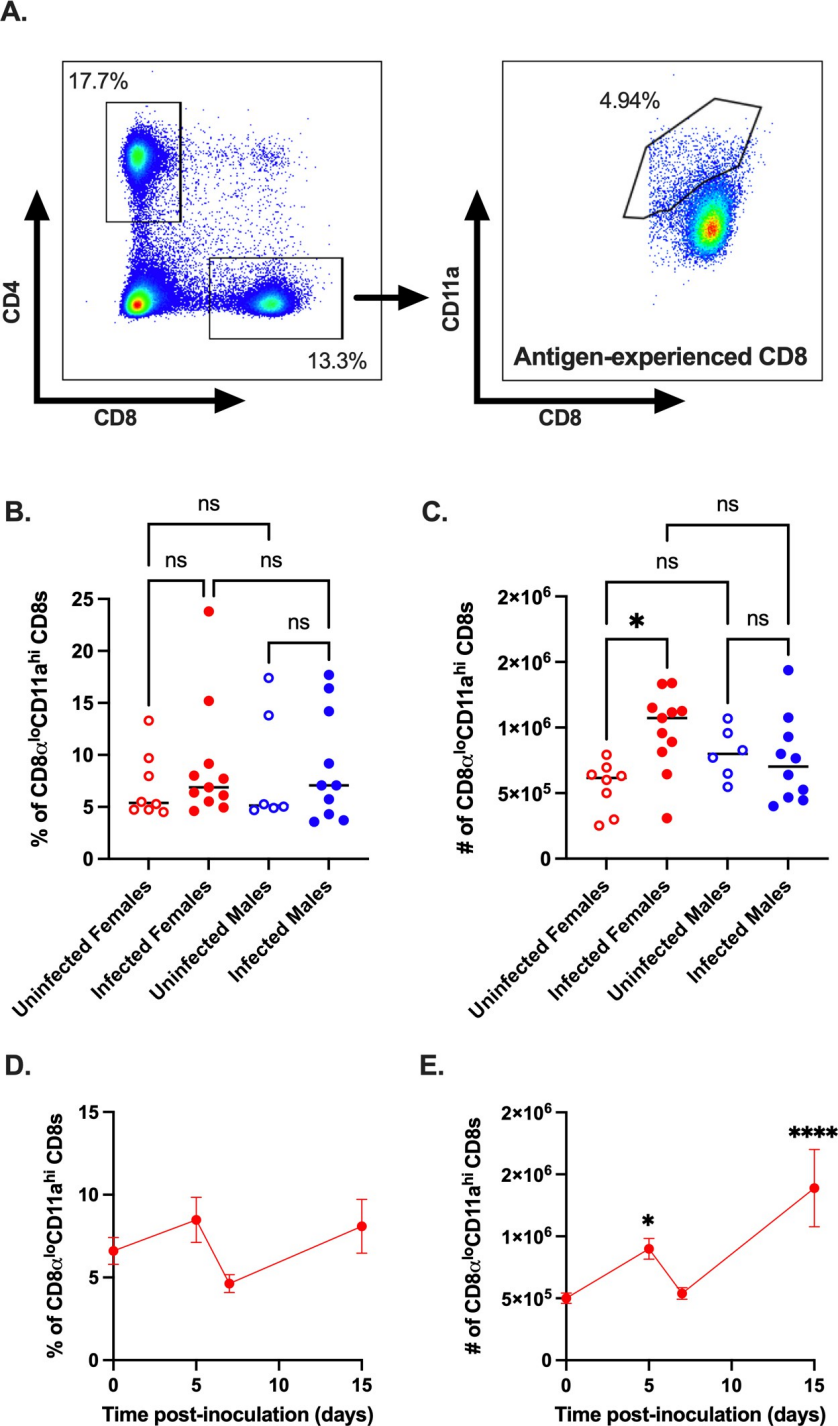
CVB3 induces expansion of antigen-experienced CD8^+^ T cells. (A) Representative flow cytometry plots for the gating strategy of CD8α^lo^CD11a^hi^ expression on CD8^+^ T cells. The frequency (B) and number (C) of CD8α^lo^CD11a^hi^ CD8^+^ T cells in male and female *Ifnar*^*-/-*^ mice 5dpi. Data points in the scatter plots represent individual mice, with lines representing the mean from two experiments. *p<0.05, One-way ANOVA. The kinetics of the frequency (D) and number (E) of CD8α^lo^CD11a^hi^ CD8^+^ T cells in female *Ifnar*^*-/-*^ mice from the spleen. All data are from two-three independent experiments with n = 7–14 mice per time point and are shown as mean ± SEM. *p<0.05, ****p<0.0001, unpaired t-test.

The downregulation of CD62L on CD8^+^ T cells can occur through bystander activation rather than direct antigen-specific engagement of the T cell receptor. Since we observed an increase in CD62L^lo^ CD8 T cells, we sought to determine if the activation of CD8 T cells in female mice was due to encountering CVB3 antigen or bystander activation. However, identifying if CVB3 generates a viral-specific CD8^+^ T cell response requires known immunodominant T cell epitopes. Unfortunately, CVB3-specific CD8^+^ T cell epitopes have yet to be clearly defined. To overcome this limitation, we examined antigen-experienced T cells using surrogate markers. Previous studies have established that antigen-experienced T cells induced following infection can be tracked, regardless of their specificity, using CD11a^hi^CD49d^+^, and CD8α^lo^CD11a^hi^ surrogate markers for CD4^+^ and CD8^+^ T cells, respectively [28,31–35]. To examine antigen-experienced CD8^+^ T cells, we analyzed the frequency and number of CD8α^lo^CD11a^hi^ CD8^+^ T cells in the spleen at 5dpi (Fig 4A). We found that the frequency of antigen-experienced CD8α^lo^CD11a^hi^ CD8^+^ T cells was not significantly different between infected and uninfected mice of either sex at 5dpi (Fig 4B). In contrast, while we did not see any difference in the number of CD8α^lo^CD11a^hi^ CD8^+^ T cells between uninfected and infected male mice, we observed a significant increase in the number of CD8α^lo^CD11a^hi^ CD8^+^ T cells in infected female mice compared to uninfected female mice (Fig 4C). These data indicate that CVB3 induces a viral-specific splenic CD8^+^ T cell response in female mice, and this T cell response can be tracked using the surrogate-maker approach.

Finally, to examine the kinetics of the antigen-experienced CD8^+^ T cell response in female mice, we orally inoculated female *Ifnar*^*-/-*^ mice with CVB3 and examined the total CD8α^lo^CD11a^hi^ CD8^+^ T cell response at 5, 7, and 15dpi. We found that while the proportion of total CD8α^lo^CD11a^hi^ CD8^+^ T cells did not change over time in response to CVB3 (Fig 4D), there were two peaks in the increase in the number of splenic CD8α^lo^CD11a^hi^ CD8^+^ T cells. At 5 and 15dpi, we found CVB3 induced a significant increase in the splenic numbers of CD8α^lo^CD11a^hi^ CD8^+^ T cells compared to uninfected female mice (Fig 4E). Further, there was a contraction in the number of CD8α^lo^CD11a^hi^ CD8^+^ T cells on day 7 post-infection. Taken together, our data indicate that CVB3 induces activation and expansion of CD8^+^ T cells at 5 and 15dpi in female mice following CVB3 infection.

### Antigen-experienced CD11a^hi^CD49d^+^ CD4^+^ T cells expand in female mice following CVB3 infection

**Fig 5:**
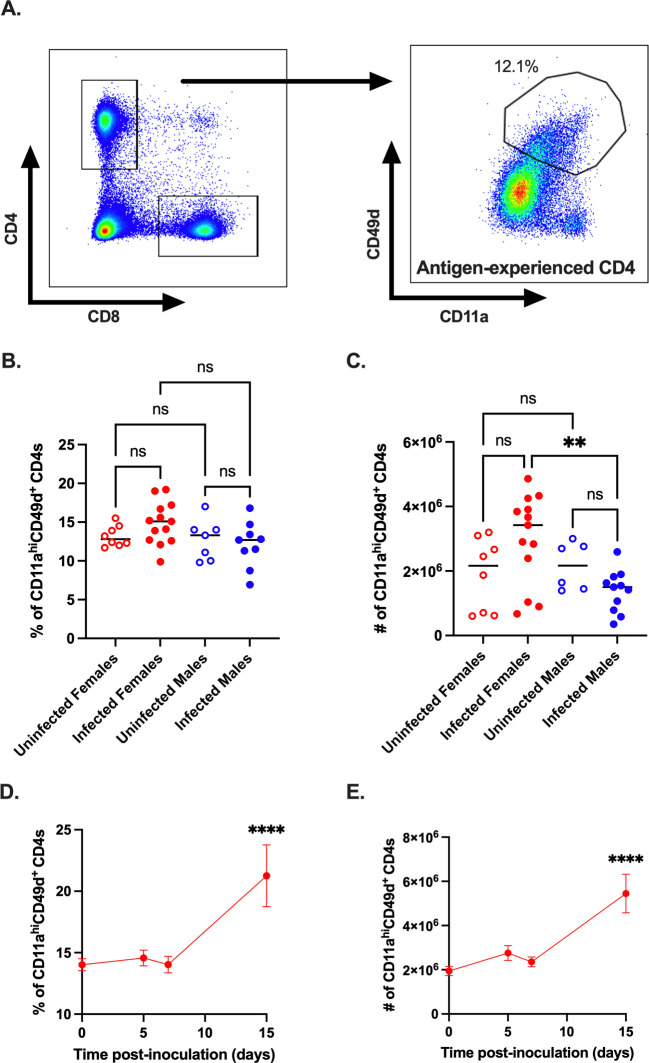
CVB3 induces expansion of antigen-experienced CD4^+^ T cells. (A) Representative flow cytometry plots for the gating strategy of CD11a^hi^ CD49d^+^ expression on CD4^+^ T cells. The frequency (B) and number (C) of CD11a^hi^ CD49d^+^ CD4^+^ T cells in male and female *Ifnar*^*-/-*^ mice 5dpi. Data points in the scatter plots represent individual mice, with lines representing the mean from two experiments. **p<0.01, One-way ANOVA. The kinetics of the frequency (D) and number (E) of CD8α^lo^CD11a^hi^ CD8^+^ T cells in female *Ifnar*^*-/-*^ mice from the spleen. All data are from two-three independent experiments with n = 7–14 mice per time point and are shown as mean ± SEM. ****p<0.0001, unpaired t-test.

CD4^+^ T cells have been previously shown to play a significant role in the CVB3-induced myocarditis [14,15,23,36]. To further characterize the T cell response in male and female *Ifnar*^*-/-*^ mice, we examined the response of antigen-experienced CD4^+^ T cells using a similar surrogate marker approach [28,31–35]. Following oral inoculation, we observed no significant difference in the frequency and numbers of CD11a^hi^CD49d^+^ CD4^+^ T cells at 5dpi in infected mice versus uninfected mice of either sex (Fig 5A—5C). However, the numbers of CD11a^hi^CD49d^+^ CD4^+^ T cells were significantly higher in infected female mice compared to infected male mice (Fig 5C). These data suggest that CVB3 infection may induce a minimal antigen-experienced CD4^+^ T cell response at 5dpi, which is higher in females than males. Next, we analyzed the CD11a^hi^CD49d^+^ CD4^+^ T cell response in female mice over time following CVB3 infection. In contrast to antigen-experienced CD8^+^ T cells, we found that the frequency and number of CD11a^hi^CD49d^+^ CD4^+^ T cells peaked at 15dpi (Fig 5D and 5E). Thus, these data indicate that CVB3 induces the expansion of antigen-experienced CD4^+^ T cells in female mice; however, this expansion occurs after the initial expansion of CD8^+^ T cells.

### Altering the inoculation route does not impact the CD8^+^ T cell response to CVB3 in female mice

**Fig 6:**
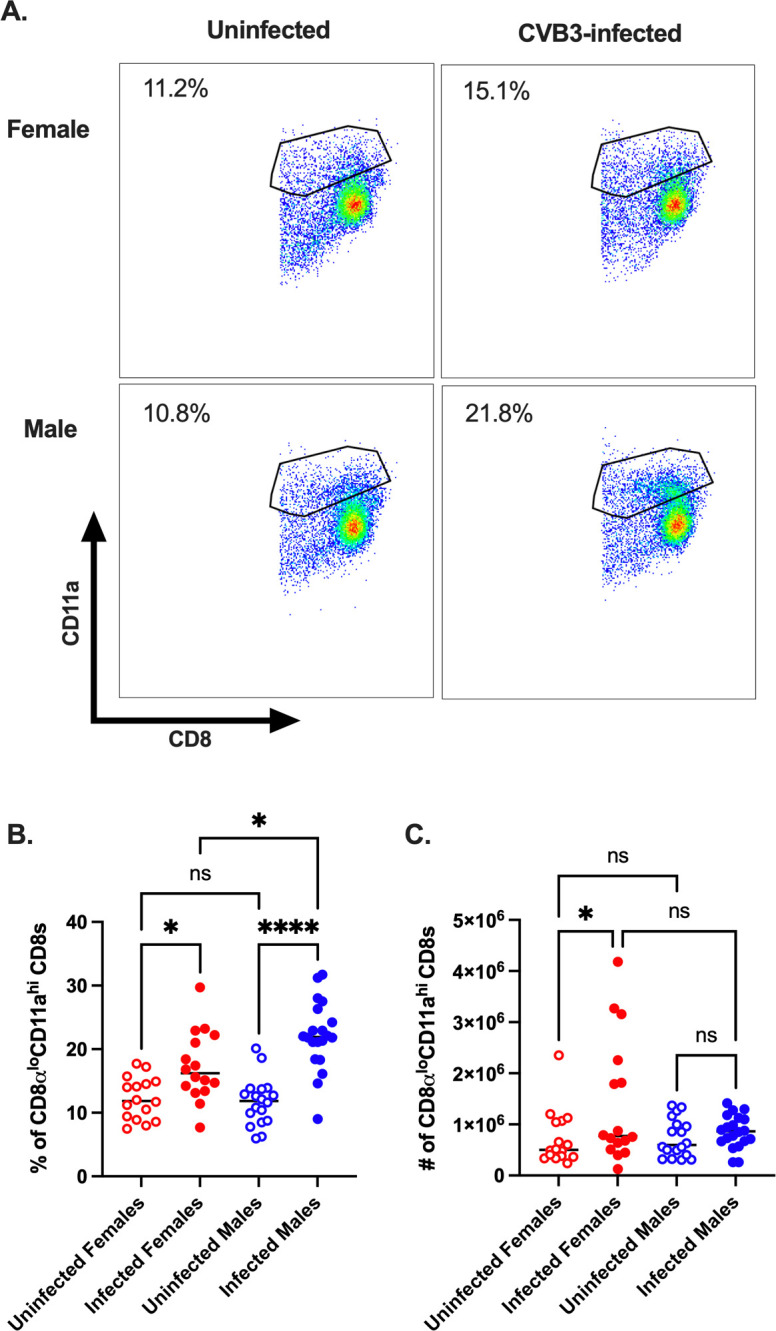
Altering the inoculation route does not impact the expansion of antigen-experienced CD8^+^ T cells in female mice. Male and female *Ifnar*^*-/-*^ mice were ip inoculated with 1x10^4^ PFU of CVB3. The spleen was harvested at 5dpi, and splenocytes were analyzed by flow cytometry. (A) Representative flow cytometry plots of CD8α^lo^CD11a^hi^ expression on CD8^+^ T cells from uninfected and infected male and female *Ifnar*^*-/-*^ mice following ip inoculation with CVB3. The frequency (B) and number (C) of CD8α^lo^CD11a^hi^ CD8^+^ T cells in uninfected and infected male and female *Ifnar*^*-/-*^ mice 5dpi. Data points in the scatter plots represent individual mice, with lines representing the mean from three experiments. *p<0.05, **p<0.01, ****p<0.0001, One-way ANOVA.

Previous studies showing a limited CD8^+^ T cell response in mice were based on a systemic infection model using intraperitoneal (ip) injections as the route of inoculation. Since the intestinal mucosa can promote robust immunity, we hypothesized that the previous lack of an observed CD8^+^ T cell response might be due to a systemic infection that bypasses the initial infection of the intestine. To investigate this hypothesis, we ip inoculated male and female *Ifnar*^*-/-*^ mice with 1x10^4^ PFUs of CVB3. At 5dpi, the spleens were harvested, and splenocytes were analyzed by flow cytometry. Similar to our oral inoculation model, we observed no significant difference in the numbers of B cells, macrophages, neutrophils, and dendritic cells between infected and uninfected male and female mice (S3 Fig). Further, we found that CVB3 infection leads to a significant decrease in the frequency of CD4^+^ T cells in male and female mice but only a significant decrease in the frequency of CD8^+^ T cells in males (Table 1). CVB3 also increased the frequency of antigen-experienced CD8^+^ T cells in male mice; however, no significant difference was observed in the number of CD8α^lo^CD11a^hi^ CD8^+^ T cells between infected or uninfected male mice (Fig 6A and 6C). In contrast to our hypothesis, we found that CVB3 increases the proportion and number of antigen-experienced CD8α^lo^CD11a^hi^ CD8^+^ T cells in infected female mice compared to uninfected female mice, similar to our findings from oral inoculation (Fig 6A–6C). These data suggest that the CD8^+^ T cell response in female mice is independent of the inoculation route.

**Table 1:** The frequency and number of splenic T cells in intraperitoneal inoculated mice.

		Frequency of cells (%) per spleen		Total number of cells (x10^4^) per spleen	
	Cell type	Uninfected	Infected	Uninfected	Infected
**Male**	CD4 T cells	19.49±0.69*	15.23±0.54*	524.91±52.98	399.04±29.64
	CD8 T cells	13.98±0.44*	11.66±0.44*	393.22±40.53	312.98±23.49
**Female**	CD4 T cells	19.44±0.71*	16.75±0.51*	501.56±38.81	540.85±70.99
	CD8 T cells	13.78±0.67	12.14±0.34	344.92±30.53	376.51±46.94

Data are presented as mean +/- SEM from two independnent experiments (n = 13–16 per group).

Significant differences between groups are denoted by asterisks (One-way ANOVA, *p<0.05).

**Fig 7:**
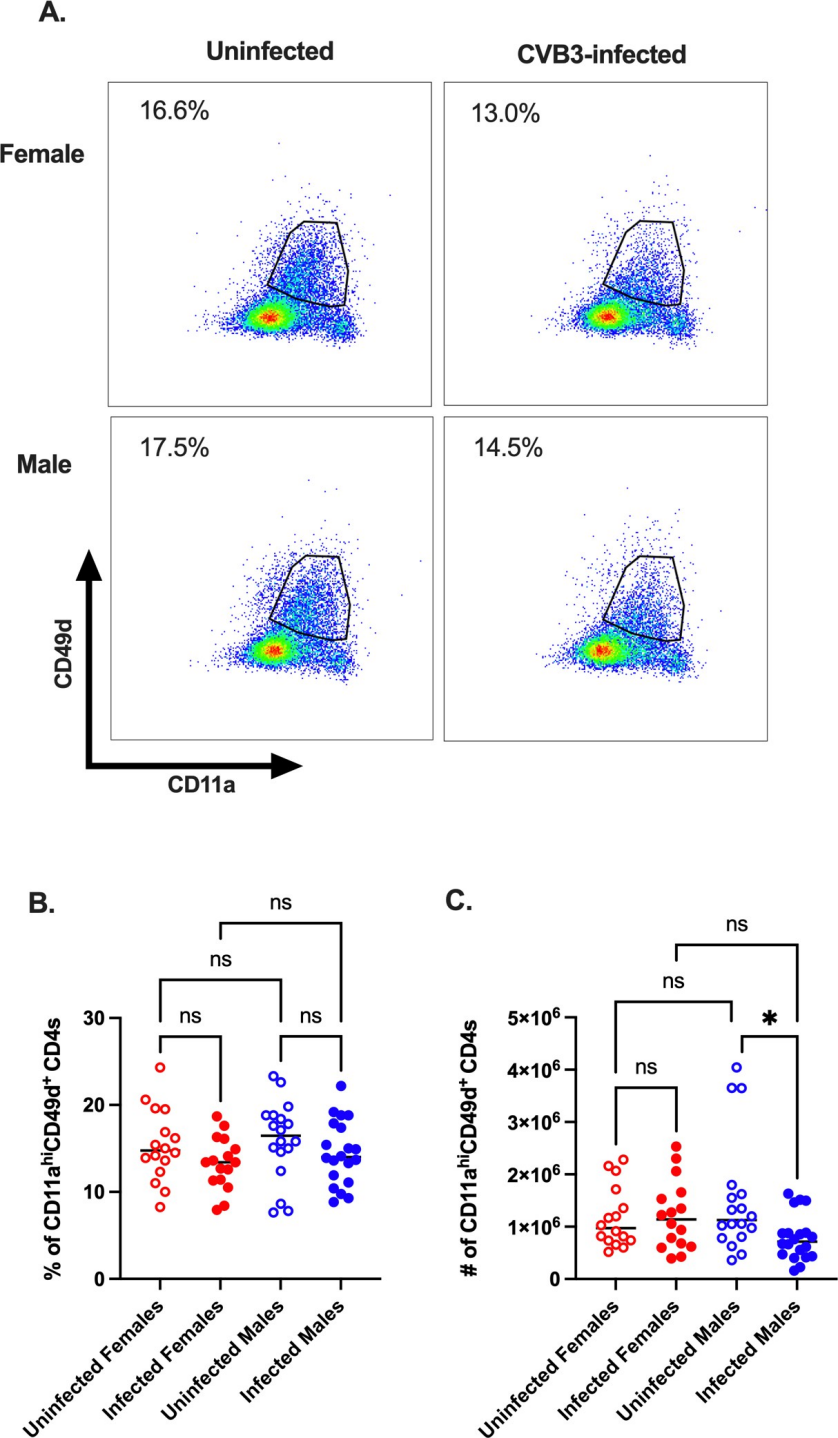
Antigen-experienced CD4^+^ T cells in CVB3-infected female mice inoculated by the ip route. Male and female *Ifnar*^*-/-*^ mice were ip inoculated with 1x10^4^ PFU of CVB3. The spleen was harvested at 5dpi, and splenocytes were analyzed by flow cytometry. (A) Representative flow cytometry plots of CD11a^hi^ CD49d^+^ expression on CD4^+^ T cells from uninfected and infected male and female *Ifnar*^*-/-*^ mice following ip inoculation with CVB3. The frequency (B) and number (C) of CD11a^hi^ CD49d^+^ CD4^+^ T cells in uninfected and infected male and female *Ifnar*^*-/-*^ mice 5dpi. Data points in the scatter plots represent individual mice, with lines representing the mean from three experiments. One-way ANOVA.

Next, to examine the CD4^+^ T cell response, we examined antigen-experienced CD11a^hi^CD49d^+^ CD4^+^ T cells. We found no statistical differences in the frequency and number of CD11a^hi^CD49d^+^ CD4^+^ T cells between uninfected and infected mice of both sexes at 5 dpi (Fig 7A–7C). Taken together, these data indicate that altering the infection route by ip injection still generates an expansion of antigen-experienced CD8^+^ T cells in female *Ifnar*^*-/-*^ mice while having little impact on antigen-experienced CD4^+^ T cells in both sexes.

### The type I interferon response does not impact the CD8^+^ T cell response in female mice

**Fig 8:**
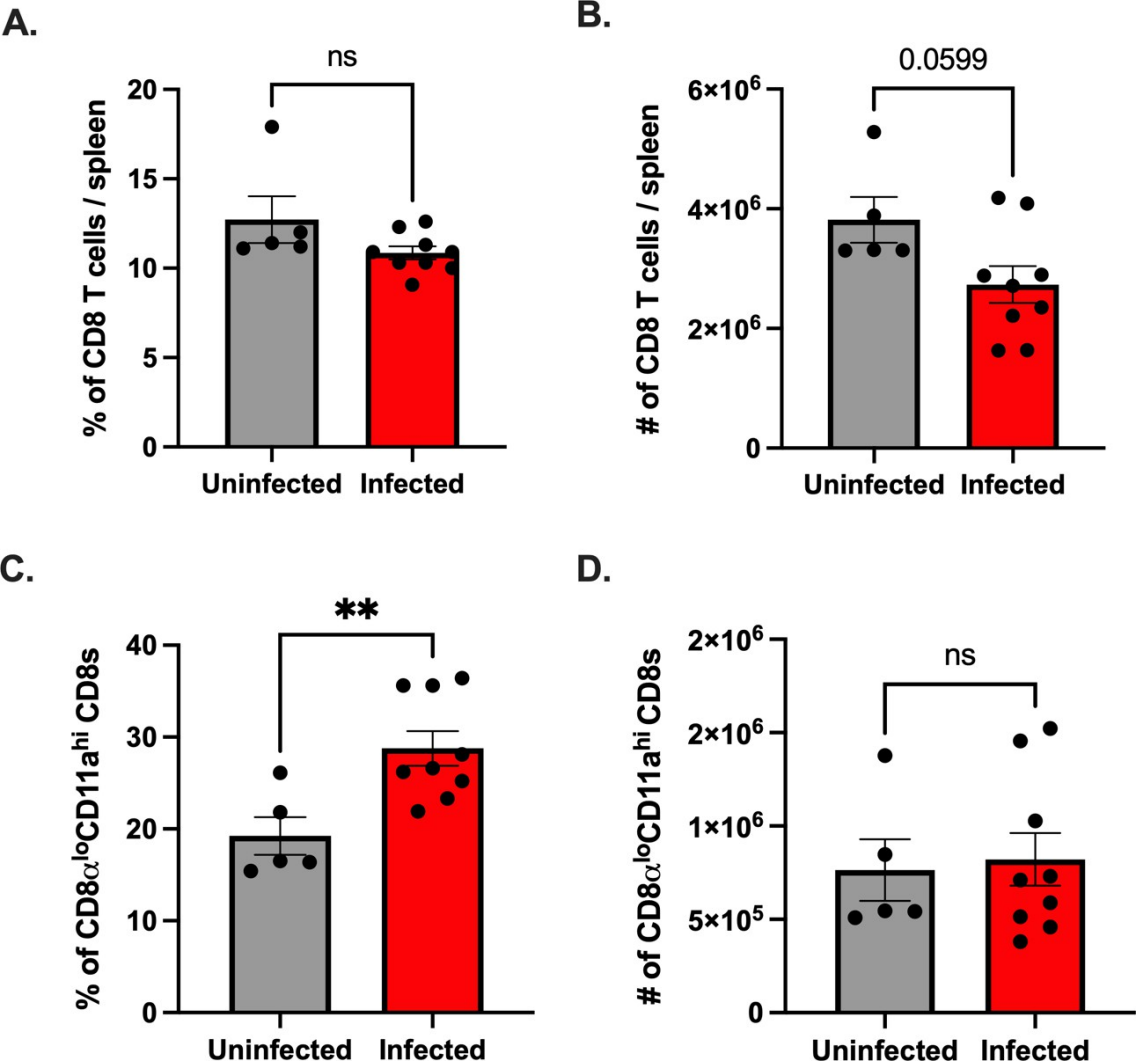
Type I IFN does not impact the expansion of antigen-experienced CD8^+^ T cells in female mice. Wild-type female C57BL/6 mice were ip inoculated with 1x10^4^ PFUs of CVB3. The spleen was harvested at dpi, and splenocytes were processed for analysis by flow cytometry. The frequency (A) and number (B) of CD8^+^ T cells in the spleen of wild-type female C57BL/6 mice 5dpi. The frequency (C) and number (D) of CD8α^lo^CD11a^hi^ CD8^+^ T cells in wild-type female C57BL/6 mice 5dpi. All data are from two independent experiments and are shown as mean ± SEM. **p<0.01, unpaired t-test.

Inflammatory cytokines, including type I interferons (IFN), have been previously shown to be necessary for the activation, proliferation, and differentiation of CD8^+^ T cells [3]. Since our data show T cell expansion in female mice that lack the type I IFN receptor, we examined the CD8^+^ T cell response in female wild-type C57BL/6 mice to determine the impact of type I IFN on this response. Following ip inoculation, we observed no difference in the frequency of splenic CD8^+^ T cells between uninfected and infected wild-type female mice (Fig 8A). However, the total numbers of splenic CD8^+^ T cells trended lower in infected female mice that reached near significance (p = 0.0599) (Fig 8B). In contrast, infected female wild-type mice had a higher frequency of CD8α^lo^CD11a^hi^ CD8^+^ T cells compared to uninfected female mice (Fig 8C). The overall number of CD8α^lo^CD11a^hi^ CD8^+^ T cells remained similar between uninfected and infected female mice (Fig 8D). However, this is likely due to the reduction in CD8^+^ T cells in infected female mice. Taken together, these data indicate that CVB3 leads to an increased proportion of antigen-experienced CD8^+^ T cells in wild-type female mice, and the loss of type I IFN does not impact this CD8^+^ T cell response.

### Antigen-experienced CD8 T cells expand due to CVB3 antigen and not due to bystander activation

**Fig 9:**
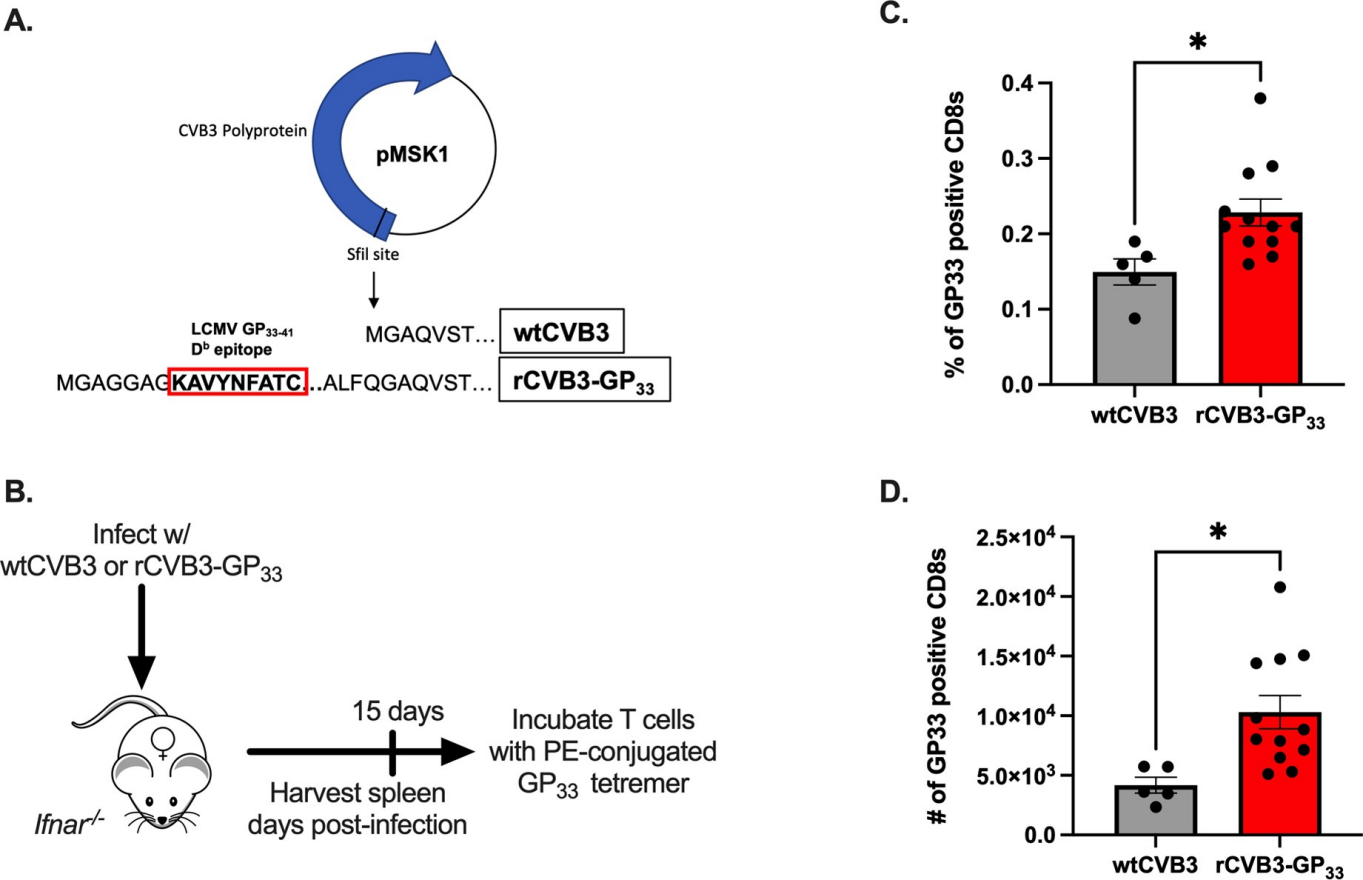
CVB3 induces a virus-specific expansion of CD8^+^ T cells in female mice. (A) the rCVB3-GP_33_ amino acid sequence encoding the LCMV GP33-41 CD8^+^ T cell epitope (adapted from [20]). (B) Schematic of the experimental design to determine the virus-specific CD8^+^ T cell response in female *Ifnar*^*-/-*^ mice. The frequency (C) and number (D) of CD8^+^ T cells staining with H2-D^b^ GP_33_ tetramer in female *Ifnar*^*-/-*^ mice 15dpi. All data are from two independent experiments and are shown as mean ± SEM. *p<0.05, unpaired t-test.

Previous studies have shown that CVB3 fails to trigger viral-specific CD8^+^ T cells *in vivo*; however, these studies have primarily been carried out in male mice [20–22]. Since we observed an expansion of CD8α^lo^CD11a^hi^ CD8^+^ T cells in female mice, we further evaluated if this CD8^+^ T cell response was viral-specific or represented the expansion of bystander CD8^+^ T cells. To address this question, we used a recombinant CVB3 (rCVB3-GP_33_) that encodes a well-characterized CD8 T cell epitope from lymphocytic choriomeningitis virus (LCMV) (Fig 9A). This recombinant CVB3, while attenuated *in vivo*, still leads to a productive infection, generating high tissue titers, and is cleared similarly to wild-type CVB3 [20–22]. Also, using the rCVB3-GP_33_ virus, no GP_33_-specific CD8^+^ T cell responses were previously detected in male mice [21]. To examine the CD8^+^ T cell response in female mice, we ip inoculated female *Ifnar*^*-/-*^ mice with 10^8^ PFU of rCVB3-GP_33_ or 10^4^ PFU of wild-type CVB3 (wtCVB3) as a control. As a control for gating GP_33_-specific CD8^+^ T cells, we also infected female *Ifnar*^*-/-*^ mice with LCMV (S4 Fig). The spleen was harvested from infected mice at 15dpi, and virus-specific CD8^+^ T cells were analyzed by flow cytometry using an H2-D^b^ GP_33_ tetramer (Fig 9B). At 15dpi, we observed a significant increase in the frequency and number of GP_33_-specific CD8^+^ T cells in the spleen from female mice infected with the rCVB3-GP_33_ virus compared to female mice infected with wtCVB3 (Fig 9C and 9D). Overall, these data indicate that CVB3 drives the expansion of virus-specific CD8^+^ T cells in female mice.

### CD8^+^ T cells protect female mice from CVB3-induced lethality

**Fig 10:**
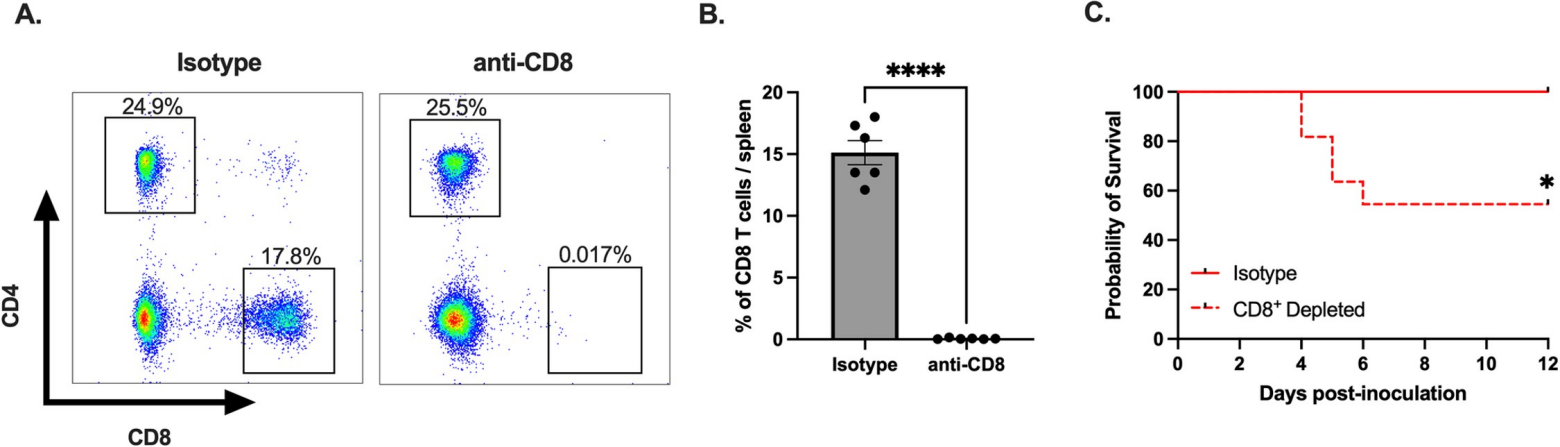
CD8^+^ T cells protect females from CVB3-induced lethality. (A) Representative flow cytometry plots of T cells gated on CD4^+^ and CD8^+^ T cell expression in female *Ifnar*^*-/-*^ mice treated with an anti-CD8α antibody or isotype control. (B) The frequency of CD8^+^ T cells in female *Ifnar*^*-/-*^ mice treated with an anti-CD8α antibody or isotype control. ****p<0.0001, unpaired t-test. (C) Survival of CVB3-infected female mice treated with anti-CD8α or isotype control antibody. *p<0.05, Log-rank (Mantel-Cox) test, n = 10–11 mice per group.

We have previously shown that female mice are protected against CVB3-induced mortality [24,26]. Since CD8^+^ T cells expand in female mice following infection, we hypothesized that these T cells might offer protection against CVB3-induced lethality. To test this hypothesis, we depleted CD8^+^ T cells with a monoclonal antibody prior to CVB3 inoculation (Fig 10A and 10B). Following inoculation with CVB3, we observed a significant increase in mortality in female mice depleted of CD8^+^ T cells compared to female mice treated with an isotype control (Fig 10C). These data demonstrate that CD8^+^ T cells play a significant role in protection against CVB3 in female mice.

## Discussion

Numerous studies have shown that sex is a significant variable in infectious diseases, and the immune response has been associated with this sexual dimorphism [1,6,37]. Similarly, sex-specific immune responses have been characterized in CVB3-induced myocarditis in mice. In this study, we examined the immune response in male and female mice following oral inoculation to identify potential immune correlates of protection. Our data indicate that female, but not male, CD8^+^ T cells are activated and respond to CVB3-specific antigen following infection. Overall, these data suggest that the CD8^+^ T cell response represents another sex-dependent immune factor during CVB3 infections.

In humans and mice, few CVB3-specific CD8^+^ T cell epitopes have been identified, and the frequency of viral-specific CD8^+^ T cells is extremely low in response to infection [38–42]. Further, mouse studies have indicated that CVB3 fails to elicit a robust CD8^+^ T cell response. Using a recombinant CVB3 that expresses the GP_33_ immunodominant peptide from LCMV, minimal differences in the CD8^+^ T cell activation markers, CD62L and CD11a, were detected. Also, a GP_33_ tetramer failed to identify any CVB3-specific CD8^+^ T cells following infection [21]. However, these conclusions were primarily drawn based on data from the infection of male mice. Our data confirm these previous findings as we found no significant indication of a detectable CD8^+^ T cell response in male mice. First, we observed a trending decrease in the frequency and number of male CD8^+^ T cells following infection in *Ifnar*^*-/-*^ mice (Fig 1D and 1E). Second, the proportion and number of antigen-experienced CD8α^lo^CD11a^hi^ CD8^+^ T cells were similar between uninfected and infected males (Fig 4B and 4C). One caveat we observed is that the proportion of antigen-experienced CD8^+^ T cells following ip inoculation of *Ifnar*^*-/-*^ males is significantly higher in infected male mice compared to uninfected male mice. However, this increase was not seen in the overall numbers, suggesting that the trending decrease in overall CD8^+^ T cells in males was the reason for the overall increase in the frequency of these CD8^+^ T cells. Finally, we previously observed that CVB3 failed to activate CD8^+^ T cells in male *Ifnar*^*-/-*^ mice with testosterone [26]. Overall, these data confirm prior studies and indicate that CVB3 fails to induce a CD8^+^ T cell response in male mice.

In contrast to males and the previous studies, our data suggest that CD8^+^ T cells expand in CVB3-infected female mice. Using a surrogate marker approach and a recombinant CVB3 expressing a well-characterized CD8^+^ T cell epitope, we found significant increases in the numbers of antigen-experienced CD8^+^ T cells (Figs 4C and 6C). Further, T cells in female mice had an activated phenotype (Fig 2A—2G), and our data indicate that CD8^+^ T cells play a significant role in protecting CVB3-infected female mice (Fig 10). Therefore, our data suggest a sex difference in the CD8^+^ T cell response to CVB3. While we did not investigate it in this study, we speculate that CVB3 elicits female CD8^+^ T cells to differentiate into effector subtypes. Examining this differentiation will be the focus of future studies.

What might explain the sex differences in the expansion of CD8^+^ T cells to CVB3? A few possibilities exist; first, the lack of CD8^+^ T cell expansion in male mice could reflect cell death of T cells due to the observed higher viral loads (S2 Fig), or the loss of naïve CD8 cells could be due to a difference in the localized response following infection. Our previous data suggest that cell death of T cells due to increased viral load is unlikely. When *Ifnar*^*-/-*^ and WT mice are infected by ip, bypassing initial infection of the intestine, we previously found similar viral loads in the peripheral tissues of male and female mice [24]; however, we still observed a similar sex difference in CD8^+^ T cell expansion (Fig 6). It is also possible that the lack of CD8 T cell expansion in the spleen of male mice may be due to a more localized response. However, our data suggest that a similar sex-dependent T cell response occurs in the MLNs (Fig 3) as compared to the spleen. Overall, these data suggest that other cell-intrinsic or cell-extrinsic factors influence the sex difference in CD8 T cell expansion following CVB3 infection.

A second possibility is that females may have an enhanced ability to prime CD8^+^ T cells in response to CVB3. Previous studies have shown that CVB3 inhibits antigen presentation by limiting the surface expression of MHC I in male dendritic cells [22]. However, antigen-presenting cells (APCs) in female mice are reported to upregulate more MHC molecules in response to stimulus than in male mice [43]. Further, the pattern-recognition receptor, toll-like receptor 7 (TLR7), is located on the X chromosome. Female dendritic cells express higher levels of TLR7, which can lead to an increased interferon response [44–46]. Since the ligand for TLR7 is single-stranded RNA that recognizes ssRNA viruses such as Coxsackievirus B3, female APCs may be inherently better at detecting and priming T cells in response to CVB3. We speculate that the ability of CVB3 to limit the expression of co-stimulatory molecules and MHC I may be restricted to males, and females may overcome this inhibition. Further studies are required to dissect the sex-specific activation and antigen presentation of dendritic cells following CVB3 infection.

Another possibility for this sex difference may be the cytokine signals required to activate CD8^+^ T cells. Type I IFNs have been identified as an important signal for CD8^+^ T cells, and the lack of type I IFN signaling can severely blunt the viral CD8^+^ T cell response [47]. Further, type I IFNs also provide a survival signal for CD8^+^ T cells during a clonal expansion [47,48]. Our data indicate that the expansion of CD8^+^ T cells in female mice is independent of type I IFN since we observed a similar expansion of female antigen-experienced CD8^+^ T cells in both wild-type and *Ifnar*^*-/-*^ (Figs 4 and 6). However, the impact of type I IFN on CD8^+^ T cell survival may explain the kinetics of CD8α^lo^CD11a^hi^ CD8^+^ T cells in CVB3-infected female mice. We observed an initial increase in the numbers of CD8α^lo^CD11a^hi^ CD8^+^ T cells in female *Ifnar*^*-/-*^ mice at 5dpi, and there was a subsequent contraction at 7dpi (Fig 4E). This may be due to the lack of IFN stimulation required for T cell survival or continued expansion. Interestingly, Wong et al. also observed similar kinetics of the contraction of splenic cytotoxic T cell activity in female, immunocompetent Balb/c mice at 7 dpi with CVB3 [49]. Our observed rebound in the numbers of CD8α^lo^CD11a^hi^ CD8^+^ T cells on day 15, also agrees with the kinetics of these previous studies. Intriguingly, Wong et al. also observed a peak of splenic cytotoxic T cells in male mice at day 7 post-infection before contracting by day 10, which may suggest sex-specific kinetic differences in the CD8^+^ T cell response to CVB3. However, it is unclear if this delay in the CD8 T cell response from male mice occurs in our model. Since male *Ifnar*^*-/-*^ mice, in our model, succumb to CVB3-induced disease around 5 dpi, we choose to focus on the T cell kinetics from female mice due to survival bias that would likely influence our results. Overall, our data indicate that CVB3 can induce the expansion of CD8^+^ T cells in female mice without type I IFN signaling, which may or may not impact the survival of these cells. Further studies are required to examine the long-term effects of type I IFN on male and female CD8^+^ T cells and if these CD8^+^ T cells differentiate into effector and memory subtypes. However, since type I IFN does not impact early expansion, our data suggest that other potential cytokines may be required to activate and lead to the expansion of CD8^+^ T cells in female mice.

Our previous studies have demonstrated an important role in type I IFN in controlling viral replication in the intestine and CVB3-induced lethality [24]. However, given that type III IFNs are important in generating an antiviral response at mucosal sites [50–52], it is interesting to speculate their possible role in the T cell response observed in our model. Studies have indicated that type III IFN can influence T cell polarization, likely through indirect interactions on antigen presenting cells [53]. Further, another study recently found that estradiol can suppress IFN-λ induced expression of interferon stimulated genes [54]; however, this suppression was cell type specific. Overall, while this suggests a potential role for type III IFNs in a sex-specific T cell response, future work is required to validate this hypothesis.

We speculate that two cytokines, interleukin-21 (IL-21) and interleukin-12 (IL-12), may serve as a possible third signal to drive CD8^+^ T cell expansion. IL-21 has been previously implicated in promoting the activation of CD8^+^ T cells during CVB3 infection. IL-21 receptor (IL-21R) knockout mice have lower CD8^+^ T cell counts following CVB3 infection. Further, infected mice with transplanted IL-21R knockout CD8^+^ T cells have less CVB3-induced myocarditis than those transplanted with wild-type CD8^+^ T cells [55]. Additionally, IL-12 has been shown to overcome the lack of type I IFN signaling on CD8^+^ T cell activation and expansion. *Ex vivo* peptide stimulation of type I IFN-receptor knockout CD8^+^ T cells in the presence of recombinant IL-12 restored clonal expansion to wild-type levels [47]. Further, *in vitro* antigen stimulation of naïve CD8^+^ T cells with IL-12 stimulated expansion and effector differentiation, similar to type I IFN [56]. Interestingly, IL-12 has also been shown to play a role in a sex-specific T cell response to *Listeria monocytogenes*. In response to bacterial infection, an enhanced capacity for CD8^+^ T cells from female mice to respond to IL-12 led to a higher proportion of short-lived effector CD8^+^ T cells [57]. In contrast, *L*. *monocytogenes* infection drove CD8^+^ T cells from male mice toward memory precursor effector cells. Further work is required to identify if IL-21, IL-12, or other cytokine signals activate CD8^+^ T cells in a sex-dependent manner and how these cytokines drive the T cell response to CVB3.

Finally, sex hormones may directly or indirectly impact CD8^+^ T cell activation, expansion, and differentiation. While differences in the absolute numbers of CD8^+^ T cells have been identified in men and women, T cells express the classical sex hormone receptors. Our lab and others have shown that sex hormones contribute to CVB3 pathogenesis. Recently we found that castrated males, depleted of endogenous testosterone, have a higher proportion and number of CD11a^hi^CD62L^lo^ CD8^+^ T cells compared to testosterone-treated males following CVB3 infection [26]. These data suggest that testosterone can impact CD8^+^ T cell activation in CVB3 infections. How testosterone and other sex hormones, such as estrogens and progesterone, directly act on CVB3-specific CD8^+^ T cells is unclear. Still, we cannot exclude the possibility that these hormones may indirectly alter the T cell response by modulating the APC or the cytokine signals.

Our data suggest that protection from CVB3 in female mice can occur due to the peripheral immune response; however, CVB3 is an enterovirus that initiates replication in the intestine. Recent data suggest that adenovirus and murine norovirus recruit Ly6A^+^CCR9^+^CD4^+^ T cells to the intestinal epithelium, which help control viral replication [58]. However, this CD4^+^ T cell response was not observed with reovirus infection, suggesting this T cell response is viral-specific. While we focused on splenic T cells in this study, the control of CVB3 replication and viral dissemination by intestinal T cells is unclear. However, our data suggest that immune correlates of protection against CVB3 may lie outside the intestine. In agreement, testosterone treatment of female mice enhances intestinal CVB3 replication, dissemination, and increases viral loads in peripheral tissues, but testosterone-treated female mice are still protected from CVB3-induced mortality. Therefore, the peripheral immune response may be necessary for controlling lethality, which may be advantageous for vaccine design.

In summary, our data yield new insight into the regulation of the CD8^+^ T cell response to CVB3 and highlight the immune differences between males and females. Our findings demonstrate that the CD8^+^ T cell response to CVB3 is sex-dependent. Future studies are required to examine if CD8^+^ T cells from female mice differentiate into effector subtypes and the ability of CVB3 to induce immune memory in these mice. Overall, these data further strengthen the idea that biological sex is an important variable that should be considered when evaluating immune responses to viral pathogens.

## Materials and methods

### Ethics statement

All animals were handled according to the Guide for the Care of Laboratory Animals of the National Institutes of Health. All mouse studies were performed at Indiana University School of Medicine (IUSM) using protocols (Approved IUSM Protocol: 20075) approved by the local Institutional Animal Care and Use Committee in a manner designated to minimalize pain, and any animals that exhibited severe disease were euthanized immediately by CO_2_ inhalation.

### Cells and virus

HeLa cells were grown in Dulbecco’s modified Eagle’s medium (DMEM) supplemented with 10% calf serum and 1% penicillin-streptomycin at 37°C with 5% CO_2_. The CVB3-Nancy and CVB3-H3 infectious clones were obtained from Marco Vignuzzi (Pasteur Institute, Paris, France) and propagated in HeLa cells as described previously [24]. CVB3 was quantified by a standard plaque assay using HeLa cells. The rCVB3-GP_33_ (rCVB3.6) was kindly provided to us by Lindsay Whitton and Taishi Kimura (Scripps Research Institute, La Jolla, California).

### Mouse experiments

C57BL/6 *PVR*^*+/+*^ wild-type and *Ifnar*^*-/-*^ mice were obtained from S. Koike (Tokyo, Japan) [59]. Mice were aged-matched and were 8–14 weeks old at the time of infection. Mice were orally inoculated with 5x10^7^ PFU CVB3-Nancy in a final volume of 20 uL. For intraperitoneal injections, mice were inoculated with 1x10^4^ PFU of CVB3 or 1x10^8^ PFU of rCVB3.6 in a final volume of 200 uL of Phosphate Buffered Saline (PBS). Data from mouse experiments were pooled from 2–3 experiments with n = 3–4 mice per group per experiment.

### Flow cytometry analysis

The spleen was collected from male and female mice at indicated time points and mechanically disrupted to generate a single-cell suspension. The erythrocytes were lysed using 1xRBC lysis buffer (BioLegend, catalog # 420301), cells were then washed and incubated with TruStain fcX (CD16/CD32. Clone 93, BioLegend, catalog # 101320), and stained with surface antibodies for indicated immune cells. Next, cells were fixed using an IC fixation buffer (Life Technologies (Fisher), catalog # 00-8222-49) and analyzed on a BD LSRFortessa flow cytometer and FlowJo software (BD Biosciences). The following mouse antibodies were used in an appropriate combination of fluorochromes: CD4 (clone GK1.5, BioLegend, catalog #100412), CD8α (clone 53–6.7, BioLegend, catalog #100725, #100711), CD8b (53–5.8, BioLegend, catalog# 140410), MHC II (clone M5/114.15.2, BioLegend, catalog #107619), CD11a (clone N418, BioLegend, catalog #117327, 101124), CD19 (clone 6D5, BioLegend, catalog #115507) (1D3/CD19, BioLegend, catalog # 152404) NK1.1 (clone PK136, BioLegend, catalog # 108706) CD11c (clone N418, BioLegend, catalog # 117327), CD40 (eBioscience (Fisher), catalog # 17040182), CD80 (clone 16-10A1, BioLegend, catalog # 104707) CD86 (clone GL-1, BioLegend, 105013), CD62L (clone MEL-14, BioLegend, catalog # 104438), and CD49d (clone R1-2, BioLegend, catalog # 103607) GP33 (Ask Stephanie), CD3e (145-2C11, BioLegend, catalog # 100306) CD69 (clone H1.2F3, BioLegend, catalog # 104521), Ly-6C (clone HK1.4, BioLegend, catalog # 128015) Ly-6G (clone 1A8, BioLegend, catalog # 127621), CD11b (clone M1/70, BioLegend, catalog # 101223) F4/80 (clone BM8, BioLegend, catalog # 123113). A representative gating strategy is provided in S5 Fig. Flow cytometry data are accessible in Immport (accession number SDY2279).

### GP_33_ tetramer staining

The H2D^b^ GP_33_ tetramer was provided as PE-conjugated by the NIH Tetramer Core facility. Spleens were collected and mechanically disrupted at 5, 8, and 15dpi to generate single-cell suspensions. Erythrocytes were lysed using 1xRBC lysis buffer, and then cells were incubated with TruStain fcX (anti-mouse CD16/CD32. Clone 93, BioLegend) for 15 mins at 1:50 dilution at 4 C and then incubated with the GP_33_ tetramer for 45 mins at 4 C at a final dilution of 1:400. Cells were washed and stained with surface antibodies for indicated immune cells.

### CD8^+^ T cell depletion

Two days before infection, 200 μg of depleting antibody (CD8 YTS 1699.4, BioXcell, catalog# BE0117) or isotype control (IgG2bκ LTF2, BioXcell, catalog # BE0090) was intraperitoneally administered to C57BL/6 *Ifnar*^*-/-*^ female mice to deplete CD8^+^ T cells. The following day tail vein blood was collected and stained to confirm CD8^+^ T cell depletion.

### Statistical analysis

Comparisons between control and study groups were analyzed using either an unpaired t-test or a one-way analysis of variance (ANOVA). Error bars in the figures represent the standard errors of the means. A p-value <0.05 was considered significant. Data were cleaned, and outliers were assessed using the ROUT method on GraphPad Prism. Power analysis was performed using the AEEC Animal Experimentation Sample Size Calculator. The estimated effect size was based on previous published studies and on pilot studies conducted in the laboratory to determine the minimum sample size of animals required to attain a statistical significance of p<0.05 and a power of 0.80 [13, 22, 35]. All analyses were performed using GraphPad Prism 9 (GraphPad Software, La Jolla, CA).

## Supporting information

S1 Fig Splenic immune cell responses in *Ifnar*^*-/-*^ mice to CVB3 following oral inoculation. Male and female *Ifnar*^*-/-*^ mice were orally inoculated with 5x10^7^ PFUs of CVB3. The frequency and number of splenic CD19^+^ B cells (A, B), neutrophils (C, D), macrophages (E, F), and monocytes (G, H). ns, not significant. *p<0.5, **p<0.01, One-way ANOVA.
(TIF)

S2 Fig CVB3 viral loads in tissues of *Ifnar*^*-/-*^ mice following oral inoculation. Male (blue) and female (red) *Ifnar*^*-/-*^ mice were orally inoculated with 5x10^7^ PFUs of CVB3. Mice were euthanized at 3 dpi (n = 6 mice per sex). All data are mean ± SEM. p<0.05, ns, not signifcant. Mann-Whitney test.
(TIF)

S3 Fig Splenic immune cell responses in *Ifnar*^*-/-*^ mice to CVB3 following ip inoculation. Male and female *Ifnar*^*-/-*^ mice were ip inoculated with 1x10^4^ PFUs of CVB3. The frequency and number of splenic CD19^+^ B cells (A, B), neutrophils (C, D), macrophages (E, F), and monocytes (G, H). ns, not significant, One-way ANOVA.
(TIF)

S4 Fig Identification of GP33-tetramer positive CD8^+^ T cells following LCMV infection. (A) Schematic of the experimental design. Female *Ifnar*^*-/-*^ mice were ip inoculated with 2x10^5^ PFU of LCMV, and at 8dpi, the spleen was harvested for flow cytometry analysis. (B) Representative flow cytometry plot for the gating strategy to identify GP33-tetramer positive CD8^+^ T cells in LCMV-infected female *Ifnar*^*-/-*^ mice.
(TIF)

S5 Fig Representative gating strategies. (A) Representative gating strategies to identify (B) B cells, (C) CD62L^lo^ CD8^+^ T cells, CD11a^hi^CD62L^lo^ CD8^+^ T cells, CD8α^lo^CD11a^hi^ CD8^+^ T cells, (D) Macrophages, (E) Neutrophils, and (F) Monocytes.
(TIF)
