# Supplementary figures and images for: Coxsackievirus B3 elicits a sex-specific CD8+ T cell response which protects female mice
Source: PLoS Pathog. 2023 Sep 5;19(9):e1011465. doi: 10.1371/journal.ppat.1011465 (PMC10503745; doi:10.1371/journal.ppat.1011465)

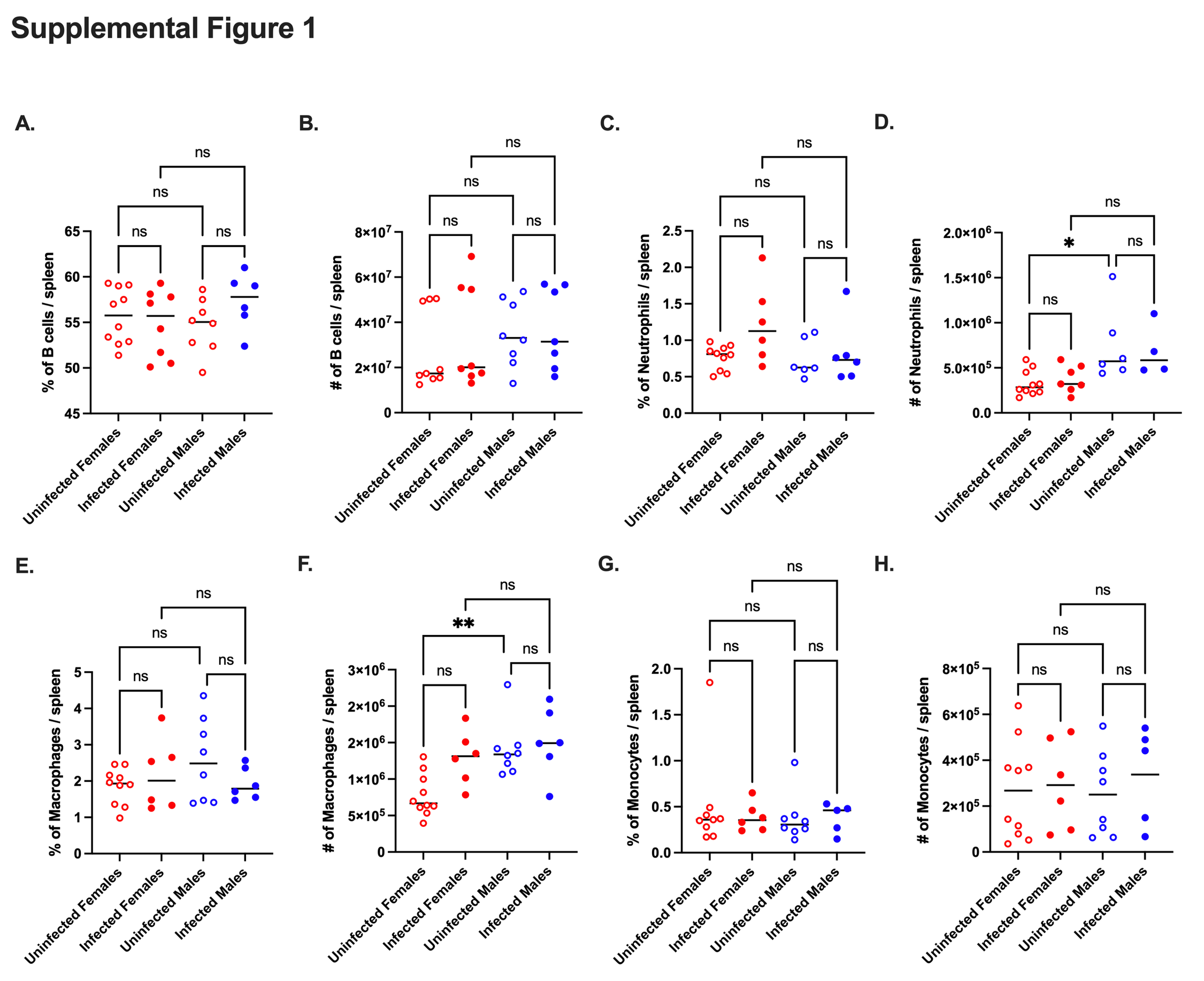

Supplement: S1 Fig — Male and female Ifnar-/- mice were orally inoculated with 5x10^7^ PFUs of CVB3. The frequency and number of splenic CD19+ B cells (A, B), neutrophils (C, D), macrophages (E, F), and monocytes (G, H). ns, not significant. *p<0.5, **p<0.01, One-way ANOVA. (TIF) [file ppat.1011465.s001.tif]

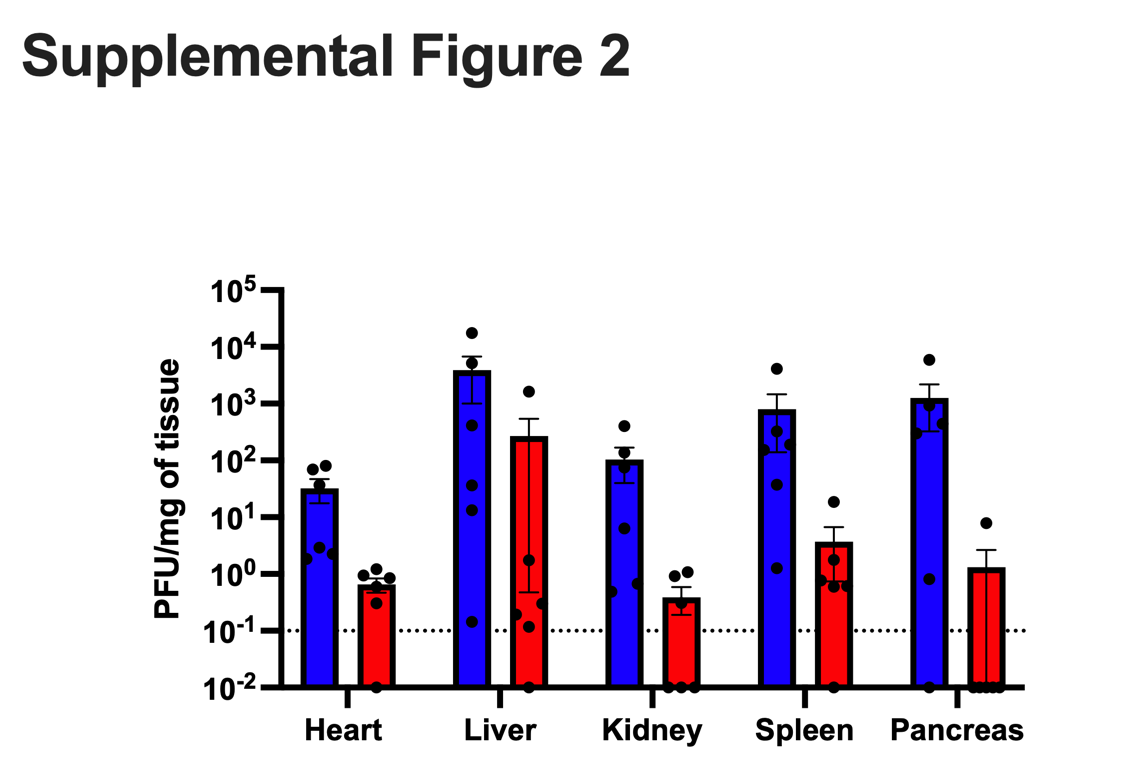

Supplement: S2 Fig — Male (blue) and female (red) Ifnar-/- mice were orally inoculated with 5x10^7^ PFUs of CVB3. Mice were euthanized at 3 dpi (n = 6 mice per sex). All data are mean ± SEM. p<0.05, ns, not signifcant. Mann-Whitney test. (TIF) [file ppat.1011465.s002.tif]

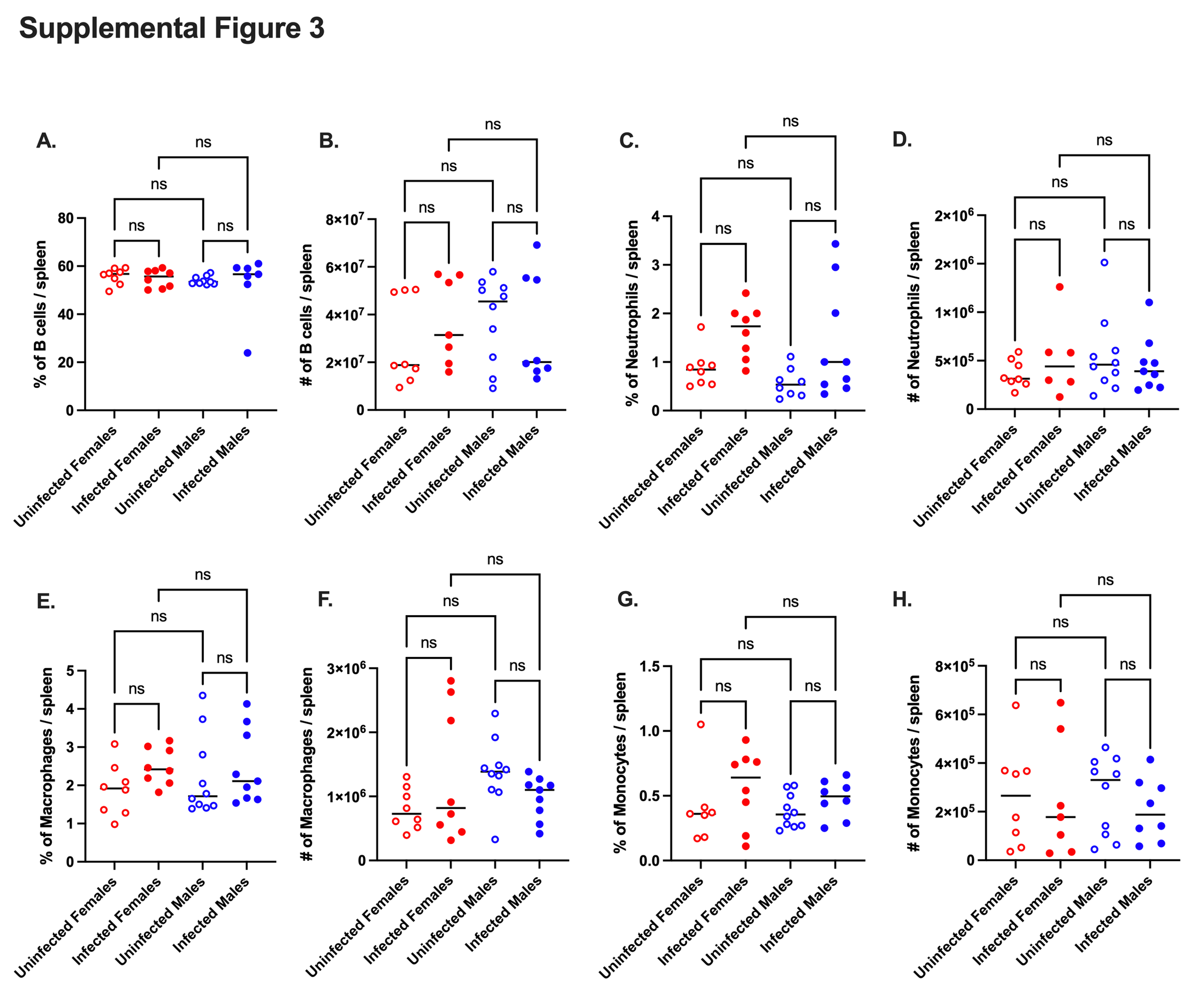

Supplement: S3 Fig — Male and female Ifnar-/- mice were ip inoculated with 1x10^4^ PFUs of CVB3. The frequency and number of splenic CD19+ B cells (A, B), neutrophils (C, D), macrophages (E, F), and monocytes (G, H). ns, not significant, One-way ANOVA. (TIF) [file ppat.1011465.s003.tif]

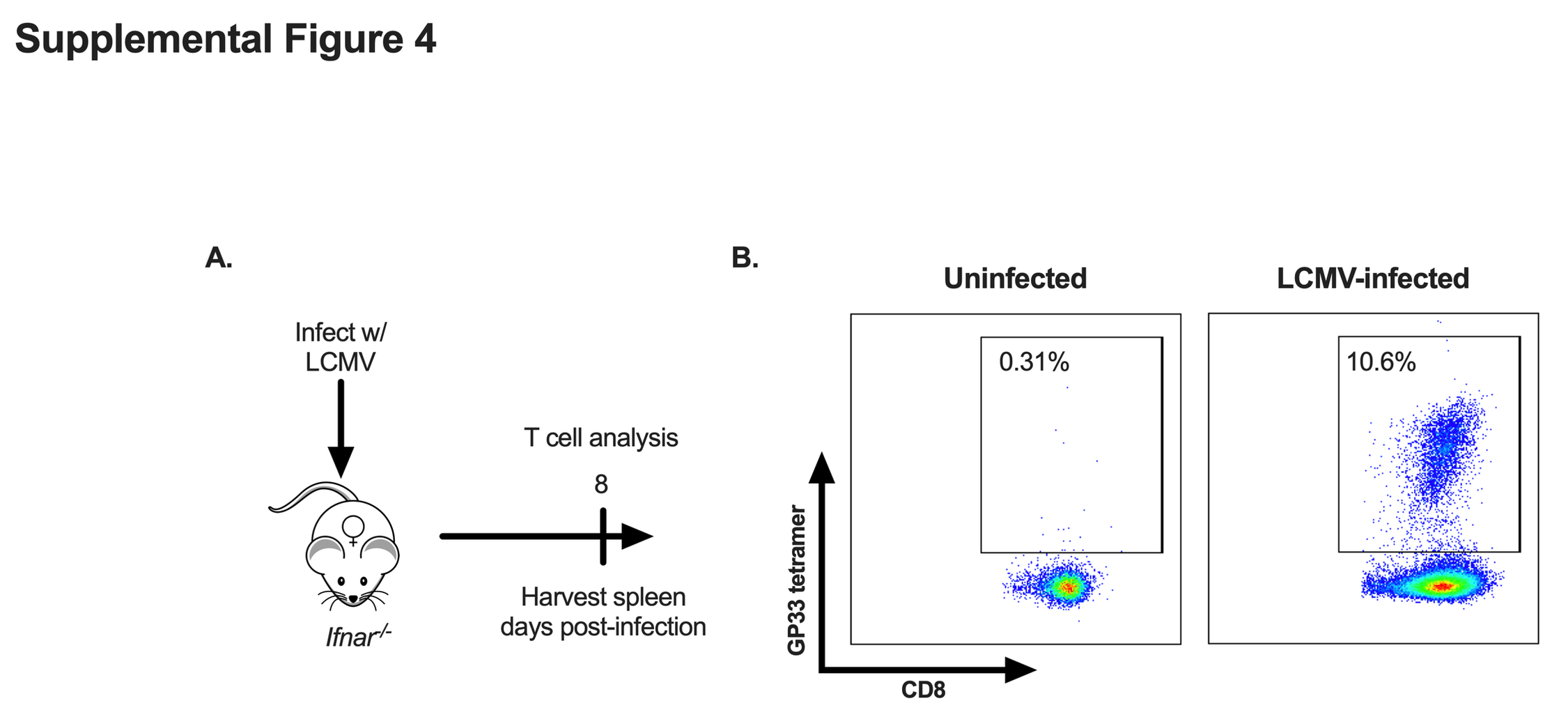

Supplement: S4 Fig — (A) Schematic of the experimental design. Female Ifnar-/- mice were ip inoculated with 2x10^5^ PFU of LCMV, and at 8dpi, the spleen was harvested for flow cytometry analysis. (B) Representative flow cytometry plot for the gating strategy to identify GP33-tetramer positive CD8+ T cells in LCMV-infected female Ifnar-/- mice. (TIF) [file ppat.1011465.s004.tif]

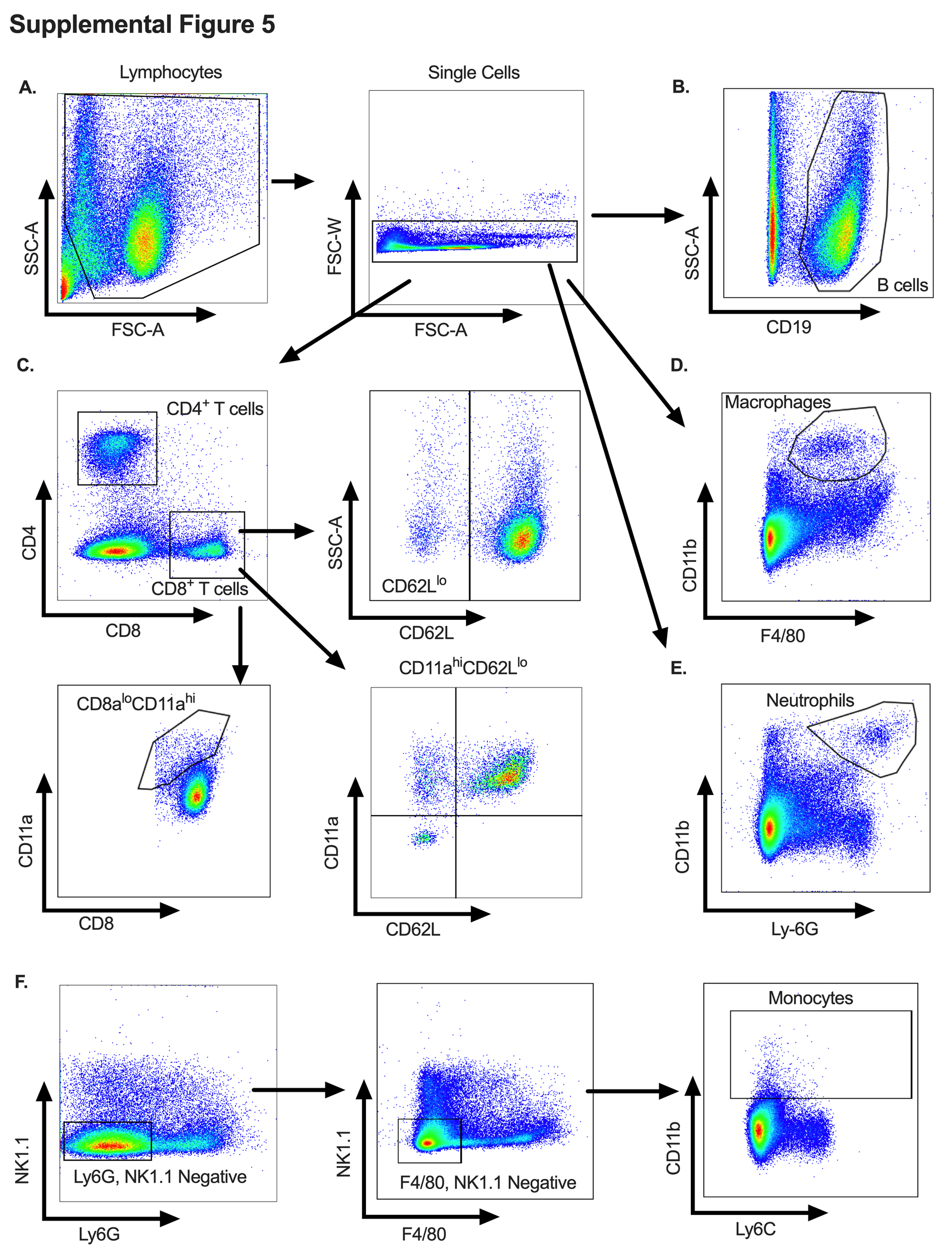

Supplement: S5 Fig — (A) Representative gating strategies to identify (B) B cells, (C) CD62Llo CD8+ T cells, CD11ahiCD62Llo CD8+ T cells, CD8αloCD11ahi CD8+ T cells, (D) Macrophages, (E) Neutrophils, and (F) Monocytes. (TIF) [file ppat.1011465.s005.tif]
